# The Surgical and Orthopædic Treatment of Infantile Paralysis

**Published:** 1884-11

**Authors:** 


### Selections.

---

### The Surgical and Orthopædic Treatment of Infantile Paralysis.

At the late meeting of the British Medical Association Bernard Roth read a paper upon this subject. In the course of his paper he says that the two guiding principles in the treatment of infantile paralysis, after the acute stage is passed, are:—

1. To improve the power of those affected muscles which have still some voluntary power left.

2. To prevent the onset of any deformity, or if this has already occurred, to reduce it to a minimum.

To carry out the indications of these principles, the first thing to be done is to correct the lowering of temperature, nearly always present, in the limb or limbs. If one leg or arm is affected you must not rest satisfied until it is as warm as the healthy one. Upon rising in the morning the patient should be sponged all over with tepid water, followed by brisk rubbing and drying. The limb must then be clothed with thick woolen goods, or, better still, with goods lined with some kind of fur. Warm baths every evening, temperature from 98 to 100° Far., are most useful. These must be followed by rapid sponging of the entire body with cold water, thus correcting the tendency to undue sensitiveness to changes in temperature. Next, *massage* or rubbing is indicated. This must be practiced regularly and more frequently than is generally recommended. Tenacious adipose tissue is often found taking the place of the wasted muscle, and through this the pressure of the rubber's hands must be transmitted before the muscle can be brought under its influence. Massage can be performed in different ways and these can be roughly classed as kneading, circular friction with the thumb, feeling, and firm stroking down. These methods can be combined with any others that may occur to the operator, the principal point being to stimulate the lowered nutrition of the muscles by artificial exercise. Besides these various motions the muscles can be exercised by putting them through their ordinary movements. As an illustration of the author's method we will quote what he says in reference to the muscles moving the shoulder joint: "The patient lying on the back, circumduction from before backwards is one of the exercises most easily taught, if there be any voluntary power, the elbow and wrist being kept extended, either voluntarily, or, if that be impossible, by means of a wooden splint. To bring the scapula muscles into action, the patient lying supine with arms down by the side of the trunk, or adjusted at right angles to the body, or extended upwards by the side of the head, is told to resist the arms being brought forward by the surgeon from either of the above positions, and then voluntarily returned to the initial position against the surgeon's gradually yielding resistance, the elbows being kept well extended. The rhomboidei and subscapularis muscles are chiefly brought into action in the second movement, with the arms at right angles to the trunk."

If the above indications are rigidly carried out, and the case has been seen early, the onset of any deformity can generally be prevented. If the case did not present itself until the deformity has occurred, it is often difficult to entirely remove it. To the suggestions above made must be added general constitutional measures. Milk should form a large element of the food. If there is any tendency to chronic constipation tepid water enemata are advised, rather than purgatives. These should be given on alternate days. The *prognosis* is favorable to some improvement within a month, if there be even so small an amount of voluntary power left, and within three months there should be decided and marked improvement. By this time the parents and friends will be properly trained, and the treatment should be continued by them as long as it is necessary. —*British Medical Journal, Sept. 6, 1884.*

